# Management of a rare, giant multicompartmental lipoma of the hand: a case report and review of the literature

**DOI:** 10.1093/jscr/rjac306

**Published:** 2022-06-28

**Authors:** Rushabh K Shah, Shakeel M Rahman, Matthew Pywell, Javier Ibanez

**Affiliations:** Department of Plastic and Reconstructive Surgery, Guy’s and St Thomas’ NHS Foundation Trust, London, UK; Department of Plastic and Reconstructive Surgery, Guy’s and St Thomas’ NHS Foundation Trust, London, UK; Department of Plastic and Reconstructive Surgery, Guy’s and St Thomas’ NHS Foundation Trust, London, UK; Department of Plastic and Reconstructive Surgery, Guy’s and St Thomas’ NHS Foundation Trust, London, UK

## Abstract

Lipomas are the commonest benign tumour, made up exclusively of adipose tissue, and can arise anywhere in the body. However, giant lipomas of the hand, defined as >5 cm in diameter, are rare. They have the potential to invade into surrounding areas and cause a multitude of symptoms due to the compression and proximity of underlying structures. We describe a case of a 64-year-old woman who presents with a swelling of the left thenar eminence, associated with numbness and tingling in all fingers. Magnetic resonance imaging and nerve conduction studies confirmed the diagnosis of a lipoma causing median nerve compression. The patient underwent elective surgical excision with good postoperative recovery. The excised lesion, measuring 12 × 7 × 2.4 cm, is one of the largest giant lipomas of the hand reported in literature, and the first to demonstrate invasion from the mid palmar space into both the dorsal sub-aponeurotic space and carpal tunnel.

## INTRODUCTION

Lipomas are the commonest benign tumour, made up exclusively of adipose tissue, and can arise anywhere in the body [1]. They most commonly affect individuals between 50 and 60 years of age, and typically present as soft, mobile and non-tender masses [2].

Giant lipomas of the hand, defined as >5 cm in diameter, are extremely rare, and tend to develop in the thenar/hypothenar spaces. They can progressively enlarge and cause symptoms of pain and nerve compression and require surgical excision due to the risk of malignant transformation [2, 3].

We describe a case of a giant, multicompartmental lipoma occurring in the left palm of a 64-year-old woman. This case is one of the largest giant lipomas of the hand reported in literature, and the first to demonstrate invasion from the mid palmar space into both the dorsum and carpal tunnel, thus representing a significant surgical challenge.

## CASE REPORT

A right hand dominant, 64-year-old woman, with a background of hypertension, was referred to our tertiary care unit with a 9-month history of progressive swelling of the left thenar eminence. She complained of reduced sensation and paraesthesia predominantly in the radial three digits, as well as a reduced range of movements of the thumb.

Physical examination revealed a non-tender, soft, palpable mass, measuring 3 × 3 cm, over the volar aspect of the left thenar eminence, with limited flexion of the interphalangeal joint of the thumb. Neurological examination demonstrated reduced sensation in the radial three and half digits of the left hand, with reduced grip strength on the ipsilateral side.

**Figure 1:**
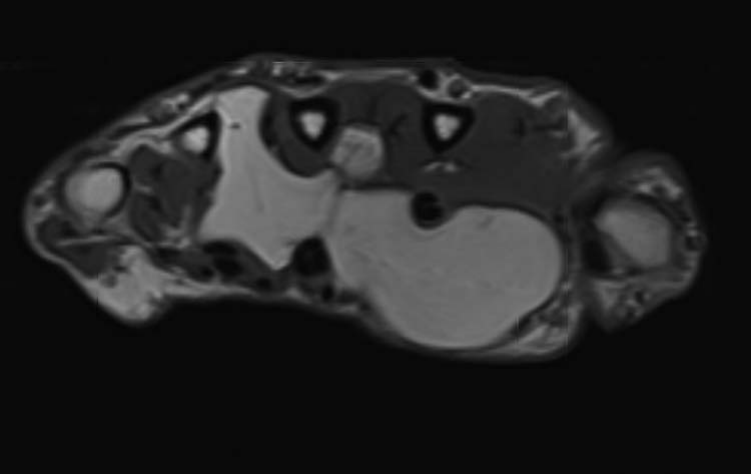
Axial MRI view demonstrating volar and dorsal extension of the fatty lesion from the middle of the palm.

**Figure 2:**
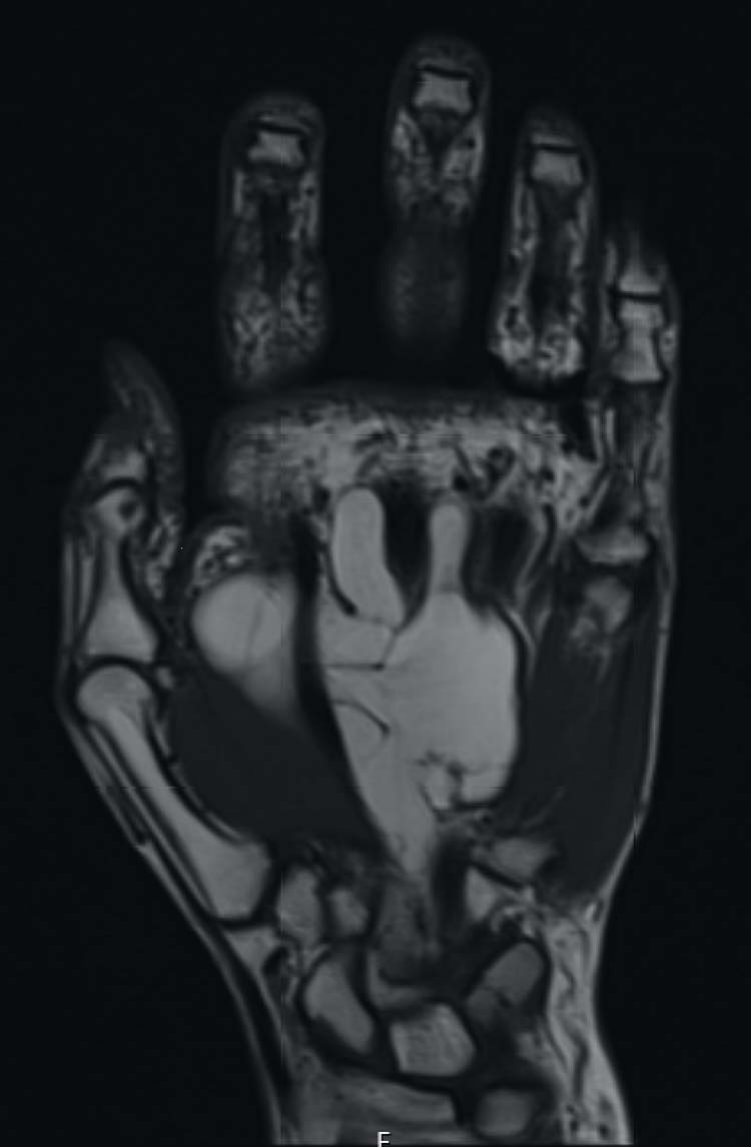
Coronal section demonstrating small proximal extension into carpal tunnel with resultant median nerve compression.

The patient underwent nerve conduction studies, which suggested a carpal tunnel lesion on the left. Magnetic resonance imaging (MRI) of the left hand confirmed the presence of a large, lobulated and well-defined fatty lesion in the palm centred between the flexor tendons and metacarpals, with dorsal and volar extensions (Fig. 1), and a proximal component within the carpal tunnel causing median nerve compression (Fig. 2). The investigations were discussed at the regional sarcoma multidisciplinary team meeting and findings were suggestive of a benign lipoma.

**Figure 3:**
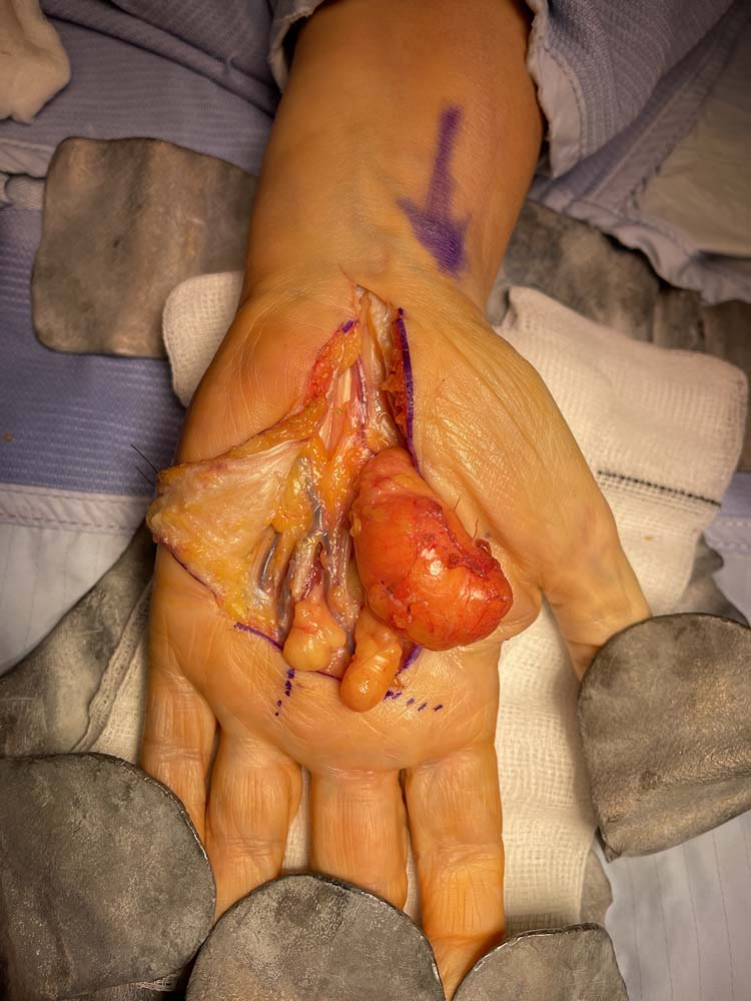
Intraoperative images demonstrating the relationship of the lesion with the surrounding neurovascular structures.

**Figure 4:**
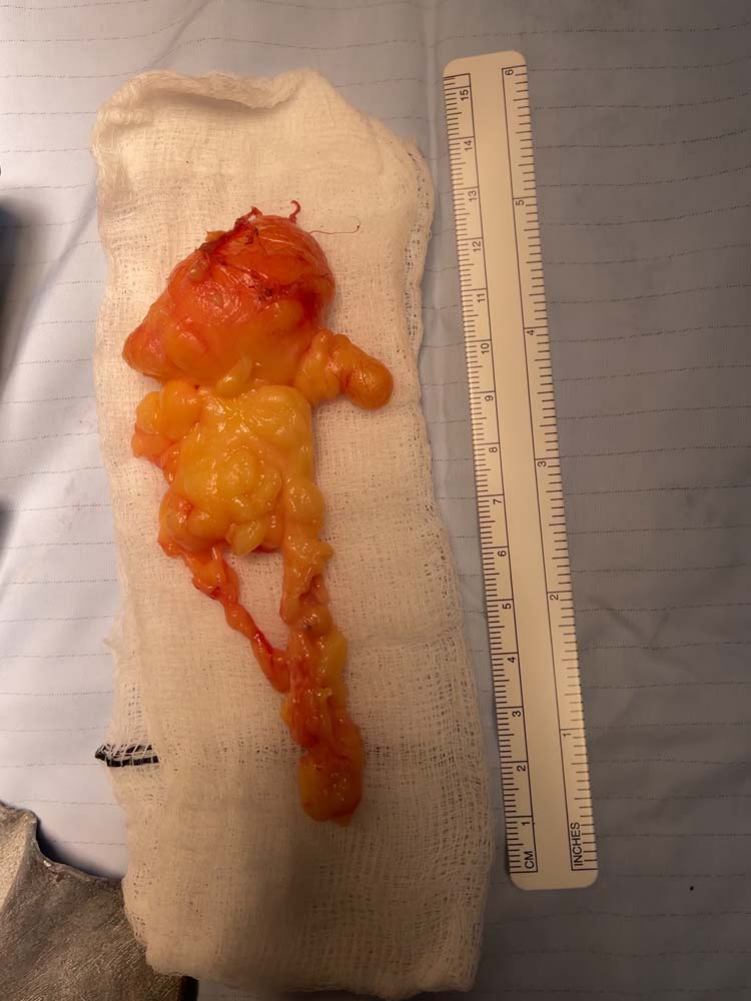
Final specimen measuring 12 × 7 × 2.4 cm.

The patient underwent an elective marginal excision under brachial plexus block and tourniquet haemostasis. A volar approach with an extended carpal tunnel release was utilized to access the lesion (Fig. 3). Given the MRI findings of the dorsal extent of the tumour, a dorsal incision was considered and marked pre-operatively. However, this was ultimately not required, as adequate access was achieved using the volar approach to excise the entirety of the lipoma. A multi-lobulated, encapsulated fatty lesion was found in the central palm extending to the carpal tunnel and the dorsal sub-aponeurotic space, involving the third inter-metacarpal space and flexor tendons. The tumour was successfully resected en bloc whilst protecting all tendons and neurovascular structures. The excised lesion measured 12 × 7 × 2.4 cm (Fig. 4). After excision, satisfactory haemostasis was achieved; drain inserted and skin closed using absorbable sutures. No immediate postoperative complications were noted.

Histopathological analysis of the soft-tissue lesion revealed sections showing lobules of mature adipocytes, consistent with a diagnosis of lipoma. There were no features of malignant change. The patient was seen in our hand dressing clinic 3 days postoperatively, whereupon the drain was removed, and wound was noted to be healing well. At the 1-month postoperative review, the patient reported complete resolution of paraesthesia with partial functional recovery including improvement in mobility of all digits and grip strength.

## DISCUSSION

Although lipomas represent the most common soft-tissue benign mass in the body, their incidence in the hand is nonetheless rare, accounting for between 1 and 3.8% of all lipomas [4]. The differentials of such a tumour include benign lesions such as ganglion cysts, giant-cell tumours and fibrolipomas of the median nerve [5]. However, the most pertinent and significant differential which must be ruled out is liposarcoma, commonly found in its well-differentiated type [6]. These commonly present with certain high-risk features; size > 5 cm, location deep to deep fascia, rapid growth and pain. When a soft-tissue tumour presents with all four clinical features, such as in our patient, there is an 86% likelihood of the mass representing malignancy, hence further investigation is mandatory to rule this out [7]. MRI provides valuable information about such lesions, and if any evidence of infiltration is seen, suspicion of malignancy is raised, and the patient must be referred for further workup at a specialist sarcoma unit [8].

Only a handful of case reports and case series have been published demonstrating nerve compression of the upper extremity due to a giant lipoma [9–12]. Our case, to our knowledge, is the one of the largest reported giant lipomas of the hand, and the first reported to demonstrate invasion into multiple compartments from the central palm, including the thenar space, dorsum and carpal tunnel resulting in median nerve compression.

The risk of malignant transformation of giant lipomas is extremely rare; hence, surgical treatment using en bloc marginal excision can provide excellent postoperative recovery, especially in cases of functional impairment including the presence of neurological symptoms [13]. Typically, palmar lipomas are removed using a volar approach that grants access to the carpal tunnel, such as the incision utilized in our case [11]. However, multicompartmental lesions may require excision through a dorsal incision, as was additionally planned in our case but not required. Such an incision dramatically reduced the risk of neurovascular lesions of the ulnar bundle and reduces the risk of tender scars over the palmar surface of the hand [14]. The predominant drawback for such approach is the high risk of fragmenting the lesion, in addition to the incomplete view of the volar compartment from the surgical field.

## CONFLICT OF INTEREST STATEMENT

The authors declare no conflict of interest and no funding.
